# Electrochemical water splitting enhancement by introducing mesoporous NiCoFe-trimetallic phosphide nanosheets as catalysts for the oxygen evolution reaction†

**DOI:** 10.1039/d4ra02344g

**Published:** 2024-05-28

**Authors:** Gouda Helal, Zhenhang Xu, Wei Zuo, Yueying Yu, Jinyan Liu, Hongping Su, Jianxin Xu, Houbin Li, Gongzhen Cheng, Pingping Zhao

**Affiliations:** a College of Chemistry and Molecular Sciences, Wuhan University Wuhan Hubei 430072 P. R. China gzcheng@whu.edu.cn; b Faculty of Science, Benha University Benha City Kalyobiya Egypt; c School of Nursing, Wuhan University Wuhan Hubei 430072 P. R. China ppzhao@whu.edu.cn; d Department of Biological and Chemical Engineering, Zhixing College of Hubei University Wuhan 430011 P. R. China liujinyan2011@163.com; e Gansu Yinguang Chemical Industry Group Co., Ltd Baiyin 730900 P. R. China

## Abstract

Transition metal-based catalysts are widely used in electrocatalysis, especially in the field of water splitting, due to their excellent electrochemical performance, which focuses on improving the efficiency of the complex oxygen evolution reaction (OER) that occurs at the anode. Transition metal-based catalysts will undergo electrochemical surface reconstruction and form (oxy)hydroxide-based hybrids, which consider the actual active sites for OER. So many efforts have been made to know the origin of the effect of electrochemical surface reconstruction on the performance of the OER. Herein, NiCoFe-phosphide catalyst nanosheets were constructed by a simple one-step hydrothermal reaction by adding oleylamine and ethanol to water solvent during the preparation of the catalyst precursor and high-temperature gas-phase phosphating and significantly showed high effectiveness catalytic activity and conductivity in comparison to normal and traditional preparation methods. Electrochemical analysis, X-ray diffraction (XRD), X-ray photoelectron spectroscopy (XPS) and high-resolution transmission electron microscopy (HRTEM) demonstrate that the surface was constructed during the electrochemical reaction and formed an amorphous layer of MO_*x*_(OH)_*y*_ active sites, which increased the electrochemical surface area and promoted charge transfer. As well, the synthesized NiCoFeP_*x*_-PNSs catalyst nanosheets exhibit excellent catalytic activity with a low overpotential equal to 259 mV to achieve the OER at a current density of 10 mA cm^−2^ and a low Tafel slope of 50.47 mV dec^−1^ which is better than for most reported transition metal-based electrocatalysts. This work provides a new design for a transition metal-based catalyst for OER as well as further insights into the effect of electrochemical surface reconstruction on intrinsic activity and OER performance.

## Abstract 2

In this study, we explord the catalytic activity of NiCoFe_phosphide nanosheets as highly active and stable catalysts for OER. Electrochemical analysis exhibits a low overpotential of 259 mV in (1 M KOH), achieving a current density of 10 mA cm^−2^ with a low Tafel slope of 50.47 mV dec^−1^.

## Graphical abstract

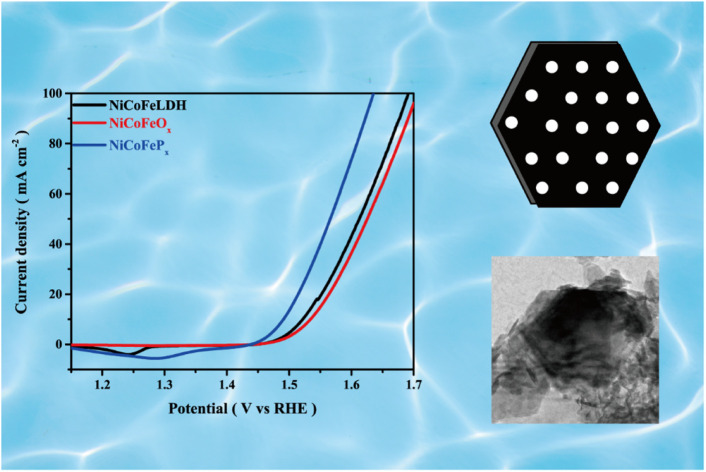

## 1. Introduction

Clean and renewable energy technologies, such as water splitting, fuel cells and metal–air batteries, are crucial for mitigating environmental pollution and the energy crisis.^1^ As an advanced energy conversion technology, water splitting is a prerequisite for the development of reversible fuel cells and rechargeable metal–air batteries.^2^ It is also considered an appealing solution to address energy-related issues without generating carbon dioxide. Electrochemical water splitting involves two half reactions: the hydrogen evolution reaction (HER) and the oxygen evolution reaction (OER).^3^ Because of the slow four-electron transfer kinetics process of OER, the application of water electrolysis is largely limited, and hence, highly effective electrocatalysts are urgently needed.^4^ At present, noble metal materials like IrO_2_ and RuO_2_ show excellent OER performance under alkaline conditions, but their high cost limits their large-scale application.^5^ To achieve highly efficient and economic electrolysis of water, it is critical to develop active, stable and cost-effective electrocatalysts for the OER, which is a thermodynamically unfavorable process.^6^ The first-row transition metals, typically Ni, Co and Fe, are highly attractive due to their low cost, relatively high conductivity, and excellent OER performance.^7^ Currently, a variety of Ni, Co and Fe transition metal-based nanomaterials (alloys, oxides, hydroxides, sulfides, phosphides and nitrides) have been reported as catalysts with extraordinary catalytic behavior of OER under alkaline conditions.^8^ Transition metal phosphides (TMPs) are particularly interesting and have attracted considerable attention, given that they exhibit intrinsically high catalytic activity and possess superior electrical conductivity.^9^ Early studies on TMPs were mainly concentrated on their applications in catalyzing the HER. Later on, TMPs with metal sites and tunable oxygen vacancies (V_O_) were found to be able to catalyze the OER under alkaline conditions.^10^ In addition, the later results showed that the starting solvents had an effect on the morphology and properties of the LDH precursors, and the properties and electrochemical activity of oxides and phosphides were closely related to the morphology and properties of their LDH precursors. The LDH precursor's structures and surface morphology with high surface area and abundant lattice defects lead to abundant active sites and confer on these materials unique properties for catalytic reactions by enhancing the kinetics of the conversion between the OER intermediates and increasing their total conductivity, which is the interest of many researchers. It was found that the LDH precursor's mesoporous nanosheet morphology can't only provide greater surface area and swifter electrolyte proximity but also a more rapid gas release rate.^11^ The starting solvents used to obtain such unique properties are of great interest and the TMPs prepared from LDH precursors with such unique properties must show superior catalytic activity toward OER. Among the TMPs, the nickel cobalt iron phosphide mesoporous nanosheets (NiCoFeP_*x*_ PNSs) have a lot of interest. One of the key advantages of NiCoFeP_*x*_ PNSs as an OER catalyst is their multifunctional nature. Each metal component contributes unique properties that enhance the overall catalytic performance of the material. For example, nickel has high electrical conductivity, which facilitates electron transfer during the OER.^12^ Cobalt has high catalytic activity and can stabilize high oxidation states, which are both important for efficient OER catalysis.^13^ Iron provides stability and corrosion resistance to the material, which are important for long-term durability.^14^ Phosphorus serves as a ligand that helps stabilize the crystal structure and enhance the electronic properties of the material.^15^ The specific composition and crystal structure of NiCoFeP_*x*_ can significantly impact its electrocatalytic activity and stability in OER. For example, several studies have shown that increasing the Co content in NiCoFeP_*x*_ can enhance its OER activity and reduce its overpotential.^16^ However, high Co content can also decrease the stability of NiCoFeP_*x*_ PNSs due to the formation of CoO_*x*_ species during OER. In contrast, increasing the Fe content can improve the stability of NiCoFeP_*x*_ but may also decrease its activity due to the lower catalytic activity of Fe compared to Co.^17^ So many efforts have been made by researchers to find a suitable structure with a suitable composition and morphology for OER catalysts. Recently, transition metal-based catalysts undergo electrochemical surface reconstruction during the OER and form oxyhydroxide-based hybrid active sites, which consider the actual-active sites and the true-catalysts for the OER. The electrochemical-based catalysts that have this ability are known as pre-catalysts.^18^ However, the mechanism for enhancing the OER performance and the correlation between the pre-catalysts and the oxyhydroxide active sites are not clear. So, many efforts have been made to understand the origin of the enhancement of the OER performance corresponding to the electrochemical surface reconstruction and to offer a design principle for the structure of these pre-catalysts.^19^ Compared with the heterostructural catalysts prepared by general methods, the heterostructures formed by the electrochemical surface reconstruction of the pre-catalysts combined with the substrate could have better electrocatalytic performance, which may be attributed to the exposure of more active sites and the optimization of the electronic structure.^20^

Herein, we create surfaces with abundant lattice defects and active sites of NiCoFe-based catalyst porous nanosheets (PNSs) as OER catalysts for water splitting and provide an insight into their electrochemical surface reconstruction during electrochemical reactions. We will discuss the synthesis approaches and structural characteristics using two different kinds of solvent mixtures, as well as their electrocatalytic performance and durability in alkaline media. For comparison, the catalysts were synthesized *via* a modification method using starting solvent mixture 1 (OAM, ethanol and water) and showed better catalytic activity than the catalysts prepared using starting solvent mixture 2 (urea, ammonium fluoride and water). The creation procedure started with the hydrothermal method to create layered double hydroxides (LDH), which undergo an oxidation process in the air to obtain the oxide, and ended by subjecting the oxide to NaH_2_PO_2_ in a nitrogen (N_2_) atmosphere, resulting in the formation of metal-based phosphide. The electrochemical analysis, XRD, XPS and HRTEM reveal that the surface was constructed during the electrochemical reaction and formed an amorphous layer of MO_*x*_(OH)_*y*_ active sites, which increased the exposing active sites, the electrochemical surface area, and promoted the charge transfer. As well, the synthesized NiCoFeP_*x*_-PNSs catalyst nanosheets exhibit excellent catalytic activity with a low overpotential equal to 259 mV in alkaline conditions (1 M KOH) to achieve the OER at a current density of 10 mA cm^−2^. These findings signify the potential of NiCoFeP_*x*_ as a practical way to enhance the activity and stability of water electrolysis, thereby optimizing the OER performance of NiCoFe-based catalysts.

## 2. Experimental section

### 2.1. Materials and chemicals

Nickel(ii) nitrate hexahydrate [Ni(NO_3_)_2_·6H_2_O, 98.0%], cobalt(ii) nitrate hexahydrate [Co(NO_3_)_2_·6H_2_O, 99.0%], iron(ii) nitrate nonahydrate [Fe(NO_3_)_2_·9H_2_O, 98.0%], ammonium fluoride [NH_4_F, ≥98.0%] and urea [CO(NH_2_)_2_, ≥98.0%] were procured from Sinopharm Chemical Reagent Co., Ltd, while oleylamine [C_18_H_37_N, 99.0%] and ethanol (C_2_H_5_OH; 99.9 wt%) were purchased from Aladdin Reagent Co. Ltd. Potassium hydroxide [KOH, ≥95%] was obtained from Alfa Aesar. The deionized (DI) water used in this study was obtained from a Millipore Autopure system with a resistivity of 18.25 MΩ cm^−1^. All other materials were of analytical grade and did not require further purification before use.

### 2.2. Synthesis of NiCoFeLDH and NiCoFeLDH-W PNSs

NiCoFeLDH PNSs were prepared by dissolving Ni(NO_3_)_2_·6H_2_O (0.15 mmol, 0.043 g), Co(NO_3_)_2_·6H_2_O (0.12 mmol, 0.035 g) and Fe(NO_3_)_2_·9H_2_O (0.03 mmol, 0.012 g) in 20 mL of DI water. Solvent mixture 1 of oleylamine (0.5 mL) with ethanol (20 mL) was added to the above solution, which was then stirred for over 30 minutes to ensure uniformity. The resulting solution was transferred to a teflon-lined stainless-steel autoclave with a capacity of 50 mL, which was sealed and heated gradually to 180 °C at a rate of 2 °C per minute. The solution was held at this temperature for 12 hours before cooling to room temperature. The resulting product was then centrifuged, washed multiple times with ethanol and ultrapure (UP) water, and dried under vacuum at 80 °C overnight. The NiCoFeLDH-W PNSs were synthesized in the same way: just replace (solvent mixture 1) by (solvent mixture 2). A solution of urea and ammonium fluoride with a molar ratio of 1 : 2 was dissolved in 20 mL of DI water and stirred until the urea was completely dissolved.

### 2.3. Synthesis of NiCoFeO_*x*_ and NiCoFeO_*x*_-W PNSs

In a typical procedure, the NiCoFeLDH PNSs and NiCoFeLDH-W PNSs were placed in ceramic boats inside a tube furnace. Then, the temperature was raised gradually (5 °C min^−1^) until it reached 350 °C, maintained there for two hours, and then lowered to room temperature. The final black products of NiCoFeO_*x*_ and NiCoFeO_*x*_-W PNSs were collected.

### 2.4. Synthesis of NiCoFeP_*x*_ and NiCoFeP_*x*_-W PNSs

In a standard process, NiCoFeO_*x*_ PNSs or NiCoFeO_*x*_-W PNSs and NaH_2_PO_2_ were acquired and subjected to the opposite ends of the ceramic boats within a tube furnace at a 1 : 10 proportion upstream ratio, all while being exposed to nitrogen gas (N_2_ flow rate of 5 sccm). The temperature was raised gradually (5 °C min^−1^) until it reached 350 °C, maintained there for two hours, and then lowered to room temperature. The final black products of NiCoFeP_*x*_ and NiCoFeP_*x*_-W PNSs were collected.

### 2.5. Electrochemical measurements

The electrochemical measurements were carried out at room temperature (30 °C) using an electrochemical workstation (CHI-760E, CH Instruments, Inc.). The electrolytic cell, consisting of a three-electrode configuration, has a glassy carbon electrode used as the working electrode; the counter electrode is made of platinum; and the reference electrode is a Hg/HgO electrode filled with 1 M KOH. The catalyst ink was prepared by dispersing 5 mg catalyst and 1 mL Nafion solution (0.1 wt%), then sonicating for 30 minutes to obtain homogenized ink. Before loading the catalyst, the surface of the glassy carbon electrode (GCE, surface area = 0.2 cm^2^) was polished with 500 nm and 50 nm α-Al_2_O_3_ successively. After being washed with ultrapure water and ethanol, 12 μL of ink was dripped on the clean surface (mass loading is 0.3 mg cm^−2^) and dried at room temperature. Linear sweep voltammetry (LSV) and cyclic voltammetry (CV) were performed to investigate the electrocatalytic performance. All voltage readings were converted to voltages *versus* the reversible hydrogen electrode (RHE) by comparing them to the corresponding reference electrode: *E* (*vs.* RHE) = *E* (Hg/HgO) + 0.92, the overpotential (*η*) was obtained from: *η* = *E* (*vs.* RHE) − 1.23 V. All the curves were recorded without *iR* correction. The Tafel plots of catalysts were depicted from their corresponding LSV data, which can be fitted based on the equation below:*η* = *a* + *b* × log(*j*), where *η* is the overpotential, *j* is the steady-state current density to the geometric area of the working electrode, and *b* is the Tafel slope. The durability test was performed using chronopotentiometry measurements at a current density of 10 mA cm^−2^. The electrochemical surface area (ECSA) was evaluated by the CV technique in the range of 1.17 to 1.27 V (*vs.* RHE) under different scan rates from 10 to 50 mV s^−1^ to avoid the generation of faradaic current. The double-layer capacitance (*C*_dl_) is linearly proportional to the ECSA, and it was estimated by plotting half of the capacitive currents at 1.22 V (*vs.* RHE) (Δ*J*/2, Δ*J* is *J*_anode_ − *J*_cathode_) against scan rate. The overall water splitting performance was assessed in a 1 M KOH solution using a NiCoFe phosphide catalyst working electrode.

### 2.6. Physical characterization

To examine the material's surface morphology and fine structure, characterization was conducted using a field emission scanning electron microscope (FESEM, Zeiss, SIGMA). The high-resolution transmission electron microscopy (HRTEM), the corresponding selected area electron diffraction (SAED) and energy dispersive X-ray spectroscopy (EDX) results were measured on the FEI Tecnai G2 F20 Field Emission Transmission Microscope. The crystal structure of the samples was revealed through X-ray diffraction (XRD, Bruker D8 Advance) X-ray diffractometer with Cu Kα radiation (*λ* = 1.54056 Å) from 10 to 70°at a scan rate of 5° min^−1^. N_2_ adsorption–desorption analysis was conducted on a Micrometrics ASAP 3020 instrument at 77 K. Thermo Fischer's ESCALAB Xi^+^ was utilized for X-ray photoelectron spectroscopy (XPS) measurement. The calibration of C 1s binding energy was set at 284.6 eV, and the data was analyzed by Casa XPS Software version 2.3.19.

## 3. Results and discussion

### 3.1. Construction and characterization of NiCoFeP_*x*_ PNSs

**Fig. 1:**
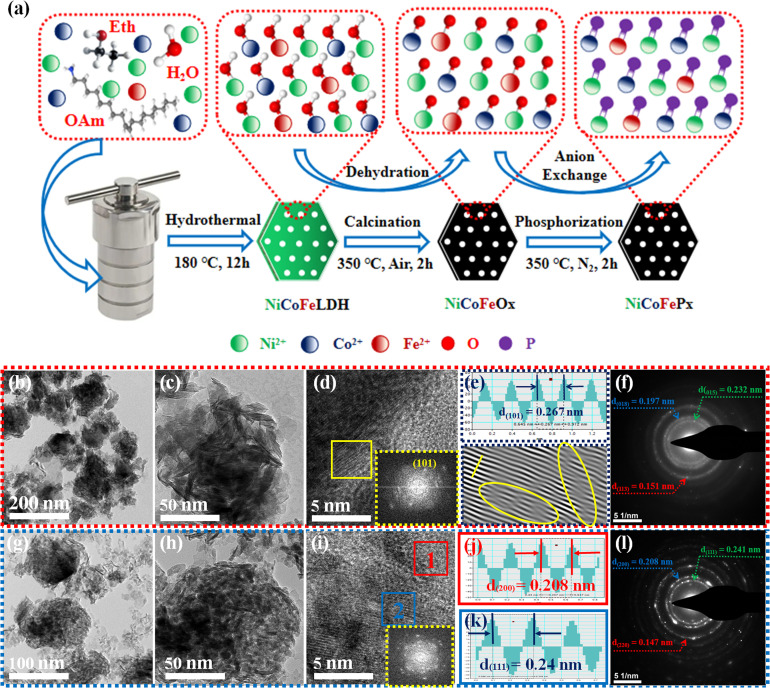
(a) Schematic diagram illustrating the synthesis process of NiCoFeP_*x*_ PNSs; morphology characterization of NiCoFeLDH PNSs: (b and c) TEM images; (d) HRTEM image (the inset is FFT image); (e) lattice images of the selected area from HRTEM; (f) SAED pattern; morphology characterization of NiCoFeO_*x*_ PNSs: (g and.h) TEM images; (i) HRTEM image (the inset is FFT image); (j and k) lattice distances of the selected areas from HRTEM; (l) SAED pattern.

**Fig. 2:**
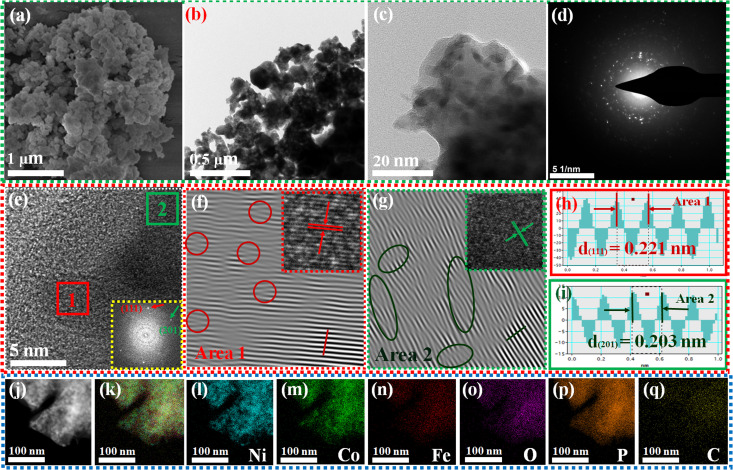
Morphology characterization of NiCoFeP_*x*_ PNSs: (a) SEM image; (b and c) TEM images; (d) SAED pattern; (e) HRTEM image (the inset is FFT image); (f and g) lattice images of the two selected areas from HRTEM; (h and i) lattice distance of the two selected areas (j) HADAF image; (k) EDX scan of main metal elements; and (l–q) element mapping.

**Fig. 3:**
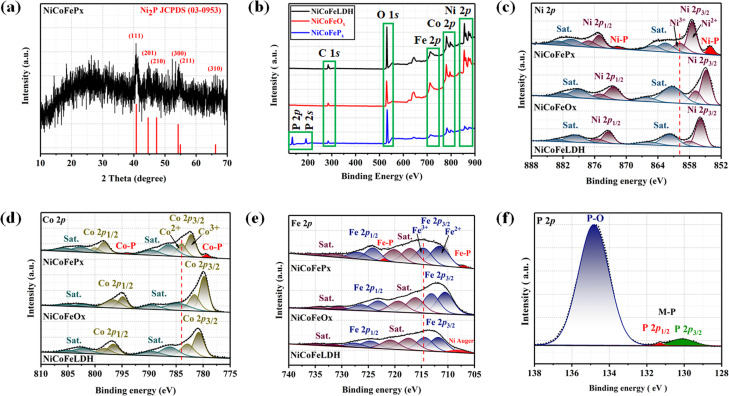
(a) XRD of NiCoFeP_*x*_; XPS fine spectra of NiCoFeLDH, NiCoFeO_*x*_ and NiCoFeP_*x*_ PNSs: (b) XPS survey spectra; (c) high resolution Ni 2p; (d) high resolution Co 2p; (e) high resolution Fe 2p; and (f) high resolution P 2p.

Fig. 1a illustrates the visual path of the synthesis of the NiCoFeP_*x*_ PNSs catalyst. Initially, NiCoFeLDH PNSs are prepared by subjecting a solution of Ni, Co and Fe salts with a solvent mixture 1 (oleylamine, ethanol and water) to a hydrothermal process at 180 °C for 12 h. Subsequently, the synthesized NiCoFeLDH PNSs undergo an oxidation process at 350 °C for 2 h in the air. The last step is the phosphorization process in a nitrogen (N_2_) environment at 350 °C for 2 h, leading to the formation of NiCoFeP_*x*_ PNSs, which facilitates the development of a surface with abundant lattice defects and active sites, thus improving the catalyst's conductivity and electrical structure. We investigated the morphologies and microstructure of the obtained samples using scanning electron microscopy (SEM) and transmission electron microscopy (TEM) imaging. The SEM images (Fig. S1, ESI†) and TEMimages (Fig. 1b, c and S2, ESI†) of the NiCoFeLDH PNSs revealed a two-dimensional (2D) morphology with ultrathin nanosheets, which indicates that the materials have a layered structure.^21^ The high-resolution transmission electron microscopy (HRTEM) imaging of one sheet of NiCoFeLDH PNSs (Fig. 1d) revealed that there are some lattice fringes in specific regions with an interplanar spacing of 0.267 nm corresponding to the crystal planes (101) of Ni(OH)_2_·0.75H_2_O (JCPDS No. 38-0715) in Fig. 1e, and we can find some of the lattice defects present on the surface. The inset FFT image (Fig. 1e) and the selected area electron diffraction (SAED) image (Fig. 1f) refer to a polycrystalline structure with ring patterns with low intensity diffraction spots, which indicates that the materials possess a low-crystallinity structure.^22^ SAED rings reveal distances of 0.151 nm, 0.197 nm and 0.232 nm, corresponding to the crystal planes (113), (018), and (015) of Ni(OH)_2_·0.75H_2_O (JCPDS No. 38-0715), respectively. The SEM (Fig. S3, ESI†) and TEM images of the NiCoFeO_*x*_ PNSs (Fig. 1g, h and S4, ESI†) revealed the same morphology as its precursor. The HRTEM image (Fig. 1i) revealed the presence of lattice fringes in some regions that have an interplanar spacing of 0.207 nm and 0.24 nm, respectively, corresponding to the crystal planes (200) and (111) of NiO (JCPDS No. 47-1049), respectively, in Fig. 1j and k. The SAED image (Fig. 1l) refers to polycrystalline materials that possess a high-crystalline structure.^23^ Some of these spots reveal distances of 0.147 nm, 0.208 nm and 0.241 nm, corresponding to the crystal planes (220), (200) and (111) of NiO (JCPDS No. 47-1049), respectively. The surface morphology of the NiCoFeP_*x*_ PNSs was studied using SEM and TEM images (Fig. 2a–c and S5 ESI†). The images confirmed 2D morphology with ultrathin nanosheets, which indicates that the materials have a layered structure, which is consistent with their precursor as layered double hydroxides. Furthermore, the nanosheets were found to have a mesoporous structure and the BET adsorption theory was used to confirm the mesoporous structure of the catalysts. The hysteresis loop in the BET curve indicated that the catalysts are a typical type IV isotherm, which reveals that the material contains a significant number of mesoporous structures.^24^ (Fig. S6–S8 and Table S1 ESI†) revealed that the BET surface areas of the NiCoFeLDH, NiCoFeO_*x*_ and NiCoFeP_*x*_ PNSs were 89.016, 122.28 and 6.51 m^2^ g^−1^, with BJH pore volumes of 0.43, 0.37 and 0.017 cm^3^ g^−1^ and small pore sizes of 14.75, 10.99 and 10.67 nm, respectively. The transformation from NiCoFeLDH to NiCoFeO_*x*_ increased the surface area exposed to PH_3_ gas, which creates the most abundant active sites on the surface of NiCoFeP_*x*_, but the addition of phosphorus can cause the metal ions to bond more strongly with each other, resulting in a more compact crystal lattice and a reduction in the surface area of NiCoFeP_*x*_. Furthermore, the pores within the material may be blocked or filled with phosphorous atoms, reducing the available surface area for adsorption. Additionally, the mesoporous structure of the nanosheets facilitates faster mass transportation during the OER process, enhancing the efficiency of the reaction.^25^ The SAED image (Fig. 2d) and the FFT image inset in Fig. 2e refer to a polycrystalline structure with low-intensity diffraction spots, which indicates that the materials possess a low-crystallinity structure,^26^ and there are no complete diffraction rings but scattered light spots, which refer to many plane phases on the surface, and there are abundant lattice defects. The HRTEM imaging of one sheet (Fig. 2e) revealed the presence of lattice fringes in certain areas (areas 1 and 2), which revealed an interplanar spacing of 0.221 and 0.203 nm corresponding to the (111) and (201) crystal planes of Ni_2_P (JCPDS No. 03-0953) in Fig. 2f–i, respectively. The two lattice images are clear evidence that the surface morphology of NiCoFeP_*x*_ PNSs has abundant lattice defects, which form from the reconstruction of atoms during the phosphorization process and facilitate the development of a surface with abundant active sites. The HAADF STEM (Fig. 2j) and elemental EDX mapping (Fig. 2k–q) show the overall distribution of Ni, Co, Fe, P, O and C elements, which prove the homogeneous distribution of the elements through the whole surface of NiCoFeP_*x*_ PNSs. On the other hand, the SEM images are used to study the morphology of the NiCoFeLDH-W, NiCoFeO_*x*_-W and NiCoFeP_*x*_-W prepared using solvent mixture 2 (Fig. S9–S11, ESI†), which revealed a 2D morphology with uniform hexagonal nanosheets. The hexagonal nanosheets observed in the images have a large size, high crystallinity and are very thick in comparison with the above ones. The crystalline structure and chemical composition were characterized by the powder X-ray diffraction (XRD) patterns. The XRD pattern of NiCoFeLDH PNSs (Fig. S12, ESI†) and NiCoFeLDH-W PNSs (Fig. S13, ESI†) showed the same diffraction peaks, which match well with Ni(OH)_2_·0.75H_2_O (JCPDS No. 38-0715). The XRD results revealed low intensity and wide peaks of NiCoFeLDH PNSs, referring to their low crystallinity and ultrathin nature in comparison with NiCoFeLDH-W PNSs. The XRD patterns of the NiCoFeO_*x*_ PNSs (Fig. S14, ESI†) and NiCoFeO_*x*_-W PNSs (Fig. S15, ESI†) matched well with NiO (JCPDS No. 47-1049).^27^ The XRD patterns of the NiCoFeP_*x*_ PNSs (Fig. 3a) and NiCoFeP_*x*_-W PNSs (Fig. S16, ESI†) show some peaks at 2*θ* = 40.79°, 44.59°, 54.23° and 66.22° with (111), (201), (300) and (310) plane phases, respectively, matched well with the Ni_2_P (JCPDS No. 03-0953).^28^ Inductively coupled plasma-optical emission spectroscopy (ICP-OES) is used to determine the elemental composition of the prepared catalysts. Based on the metal ion ratio obtained from the ICP-OES analysis, the NiCoFe-based samples can be denoted as Ni_5_Co_3.80_Fe_1_LDH, Ni_5_Co_3.84_Fe_1.02_O_*x*_, and Ni_5_Co_4.08_Fe_1.03_P_*x*_ PNSs, respectively. The results were very close to their feeding ratio, as shown in Table S2, ESI.† Furthermore, the EDX mapping ratio shows that the metal ion ratio of NiCoFeP_*x*_ PNSs is very close to the feeding ratio, as shown in Fig. S17, ESI.†

### 3.2. Electronic structure and valence state analysis

The electronic structure of the synthesized catalysts was analyzed using X-ray photoelectron spectroscopy (XPS) measurements. The XPS survey spectra (Fig. 3b) confirm the presence of Ni, Co, Fe and O in all three samples. Moreover, two additional peaks of P 2p and P 2s were detected for the NiCoFeP_*x*_ PNSs, indicating the successful formation of NiCoFeP_*x*_ PNSs. The absence of any unexpected peaks in the survey spectra indicates that the synthesized catalysts are pure and free from any impurities or contaminants. The Ni 2p XPS profile (Fig. 3c) shows a spin–orbit doublet of Ni 2p_1/2_ and Ni 2p_3/2_, as well as two satellite humps next to the peaks, which refer to the formation of Ni–PO_*x*_.^29^ The presence of Ni^3+^ and Ni^2+^ indicates that Ni ions are partially oxidized, can participate in redox reactions and allow for the efficient transfer of electrons during the electrocatalytic process, which is important for their catalytic activity. The observation of two small peaks of Ni–P suggests the involvement of phosphorus in the catalyst's formation to form nickel phosphide. The slightly higher binding energy observed in the Ni 2p of NiCoFeP_*x*_ PNSs compared to the other synthesized catalysts indicated that they were beneficial for more Ni^3+^ active species and promoted the electrocatalytic process. The Co 2p XPS spectrum (Fig. 3d) also shows a two-spin orbit doublet of Co 2p_1/2_ and Co 2p_3/2_, along with two satellite humps next to the peaks, referring to the formation of Co–PO_*x*_.^30^ The presence of both Co^3+^ and Co^2+^ species suggests that the Co ions are partially oxidized and can participate in redox reactions during the electrocatalytic process. Also, two small peaks of Co–P are observed, suggesting the involvement of phosphorus in the catalyst's formation to form cobalt phosphide. The slight shift to high binding energy observed for NiCoFeP_*x*_ PNSs suggests a beneficial formation of Co^2+^ active species during the electrocatalytic process.^31^ The Fe 2p XPS spectrum (Fig. 3e) shows a two-spin orbit doublet of Fe 2p_1/2_ and Fe 2p_3/2_, along with two satellite humps referring to the formation of Fe–PO_*x*_.^32^ These peaks, along with the presence of Fe^2+^ and Fe^3+^ species and satellite peaks, suggest that the synthesized catalysts have a complex electronic structure, which can play a crucial role in their electrocatalytic activity. The Fe 2p XPS spectra of NiCoFeLDH PNSs show the auger peak of Ni^2+^. Additionally, the NiCoFeP_*x*_ PNSs show very small peaks corresponding to the formation of iron phosphide (Fe–P). The P 2p XPS spectrum (Fig. 3f) displays three peaks, which correspond to phosphate and phosphide peaks (P–O and M–P_3/2_, M–P_1/2_), respectively.^33^ The detection of P–O (phosphates PO_4_^3−^ or PO_3_^−^ or P_2_O_5_) peaks suggests that the metal phosphide has undergone oxidation, leading to a higher degree of oxidation probably caused by the exposure of phosphide species to air, which can further enhance the catalytic activity of the synthesized catalysts.

### 3.3. Electrochemical performance of NiCoFeP_*x*_ PNSs and comparison samples

**Fig. 4:**
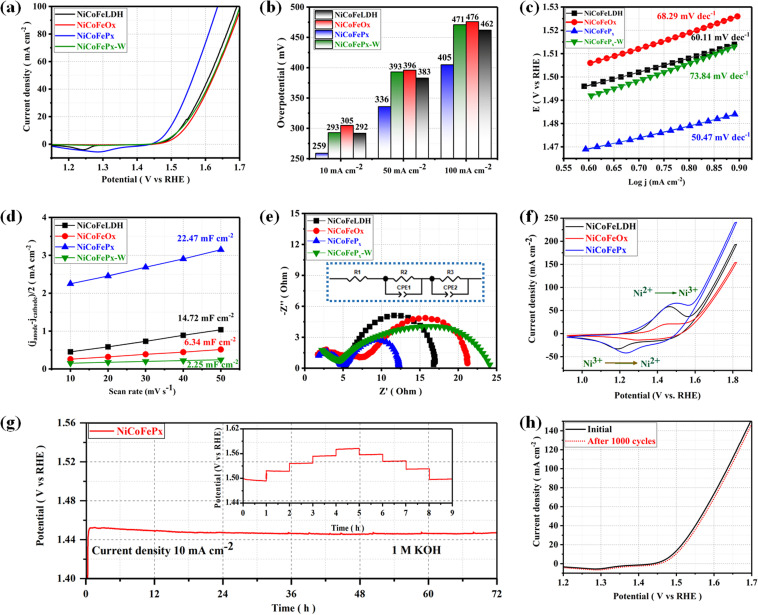
Electrochemical measurements result: (a) LSV curves of OER; (b) overpotential histogram at 10, 50 and 100 mA cm^−2^; (c) Tafel plots; (d) the capacitive current at 1.22 V *vs.* RHE as a function of the scan rate; (e) Nyquist plots measured at 0.6 V *versus* Hg/HgO (inset equivalent circuit diagram); (f) CV curves in the potential range from 0.9 to 1.8 V. (g) Chronopotentiometry of the NiCoFeP_*x*_ at a current density of 10 A cm^−2^ for OER (the inset is durability at different current density); (h) LSV curves of NiCoFeP_*x*_ PNSs before and after multiple cycles stability.

The electrocatalytic performances of the as-prepared samples were investigated in a 1 M KOH solution using a typical three-electrode system. The linear sweep voltammetry (LSV) is used to investigate the electrocatalytic activity of materials for OER at a scan rate of 5 mV s^−1^ with a working electrode connected to the rotating disk electrode system and rotated at 1600 rpm. The temperature of the entire system was maintained at 30 °C. Starting with the catalyst prepared using solvent mixture 1, the LSV curves (Fig. 4a) show that the NiCoFeP_*x*_ PNSs exhibited the best catalytic activity with the lowest overpotential at 10 mA cm^−2^ (*η*_10_ = 259 mV), which is better than NiCoFeLDH PNSs (*η*_10_ = 292 mV) and NiCoFeO_*x*_ PNSs (*η*_10_ = 305 mV). This result indicates that the introduction of a proper amount of P can effectively improve the electrocatalytic OER activities of the catalysts, which may be caused by forming, exposing more active sites, abundant vacancies and defects that accelerate the charge transfer between the intermediates and the active sites and require less energy to drive the reaction compared to the other prepared catalysts. The overpotential plot diagram of the as-synthesized samples at current densities of 10, 50 and 100 mA cm^−2^ shows that the electrocatalytic behavior of NiCoFeP_*x*_ materials is significantly better than the other samples (Fig. 4b). The OER kinetics were evaluated by the Tafel slope derived from the LSV curves. As shown in Fig. 4c, NiCoFeP_*x*_ PNSs present a Tafel slope (50.47 mV dec^−1^), which is much smaller than NiCoFeLDH, (60.11 mV dec^−1^) and NiCoFeO_*x*_ PNSs (68.29 mV dec^−1^), indicating that the P doping can improve the reaction kinetics and accelerate the rate of the OER. The intrinsic properties of the electrocatalytic surface of the prepared samples were investigated using electrochemical double-layer capacitance (*C*_dl_). As shown in Fig. 4d, the *C*_dl_ value of NiCoFeP_*x*_ PNSs is (22.47 mF cm^−2^) which is higher than NiCoFeLDH PNSs (14.72 mF cm^−2^) and NiCoFeO_*x*_ PNSs (6.34 mF cm^−2^). The results suggest that the NiCoFeP_*x*_ PNSs have a higher electrochemically active surface area, which is closely related to the density of active sites on the surface, which reflects the reason for the high catalytic activity of NiCoFeP_*x*_ PNSs. Electrochemical impedance spectroscopy (EIS) was utilized to investigate the electrochemical dynamics of charge transfer between the working electrode, catalyst and electrolyte at a potential of 1.52 V (*vs.* RHE). The Nyquist plots of all the samples were fitted to determine the charge transfer resistance (*R*_ct_). The semicircle diameter (Fig. 4e) obtained from the EIS analysis is related to (*R*_ct_). The results showed that the NiCoFeP_*x*_ PNSs have the smallest Nyquist fitting semicircle diameter (*R*_ct_ = 7 Ω), compared to NiCoFeLDH PNSs (*R*_ct_ = 11.7 Ω), and NiCoFeO_*x*_ PNSs (*R*_ct_ = 14 Ω) which refer to low impedance during charge transfer due to the high ECSA and high density of OER active sites on the surface, which can accelerate the rate of the OER and decrease the overpotential. To know the surface reconstruction during the OER and the main metal active sites on the surface of the catalyst, the CV curves in the potential range from 0.9 to 1.8 V were studied. Fig. 4f shows a redox peak around 1.49 V, corresponding to the Ni^2+^/Ni^3+^ redox couple. CV curves clearly show that Ni is the main metal active site on the surface, and the NiCoFeP_*x*_ PNSs have a stronger Ni^2+^/Ni^3+^ electrochemical response than the other catalyst, indicating that the introduction of the P element during the catalyst preparation could create more electroactive sites on the surface. The long-term electrochemical durability of the NiCoFeP_*x*_ PNSs catalyst was studied by a chronopotentiometric test, as shown in Fig. 4g. The results exhibit excellent long-term stability, with no significant degradation observed during the 72 hour test period. The multi-step chronopotentiometry test is used to further approve the electrocatalytic durability at different current densities from 10 to 50 mA cm^−2^, with a change of 10 mA cm^−2^ per hour (inset image, Fig. 4g). The results showed relatively stable current platforms at changing current densities, indicating the great electrochemical universality and high conductivity characteristics of the NiCoFeP_*x*_ PNSs. To further approve the durability, the LSV curves before and after 1000 continuous CV cycles are also compared (Fig. 4h). The LSV curve after the cyclic test remained almost unchanged, suggesting that the NiCoFeP_*x*_ PNSs are a promising candidate for practical applications in various electrochemical processes.

On the other hand, the catalytic activity of the catalysts prepared using solvent mixture 2 was studied in Fig. S18, ESI.† The results showed that the properties and electrochemical activity of oxides and phosphides are closely related to the morphology and properties of their LDH precursors. The NiCoFeP_*x*_-W PNSs exhibited the best catalytic activity, with the lowest overpotential of (*η*_10_ = 293 mV), Tafel slope (73.84 mV dec^−1^), and EIS (*R*_ct_ = 19 Ω), with stability reaching 24 h at a current density of 10 mA cm^−2^. The results showed that the catalytic behavior of NiCoFeP_*x*_ PNSs is better than that of NiCoFeP_*x*_-W PNSs, reflecting the advantage of adding oleylamine and ethanol to the water solvent during catalyst preparation. Some of these advantages include: Firstly, oleylamine is a surfactant that helps in dissolving metal precursors such as nickel, cobalt and iron salts in ethanol and ensures that the metal precursors are uniformly dispersed in the solution, leading to a homogeneous catalyst. Secondly, oleylamine acts as a stabilizing agent, preventing the aggregation or precipitation of metal ions during the synthesis process, which ensures that the resulting catalyst has a high surface area and well-defined morphology, which is crucial for efficient catalytic activity. Thirdly, by controlling the particle size of the NiCoFeLDH catalyst by adjusting the concentration or ratio of these components, it is possible to obtain nanoparticles with the desired sizes, which can significantly influence the catalytic performance.

### 3.4. Insights into electrochemical surface reconstruction based on HRTEM, XRD and XPS

**Fig. 5:**
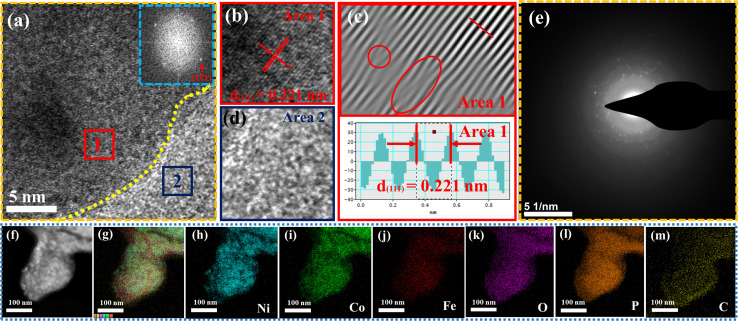
Morphology characterization of NiCoFeP_*x*_ PNSs after OER: (a) HRTEM image (the inset is FFT image); (b–d) lattice images of the two selected areas from HRTEM; (e) SAED pattern; (f) HADAF image; (g) EDX scan of main metal elements; and (h–m) element mapping.

**Fig. 6:**
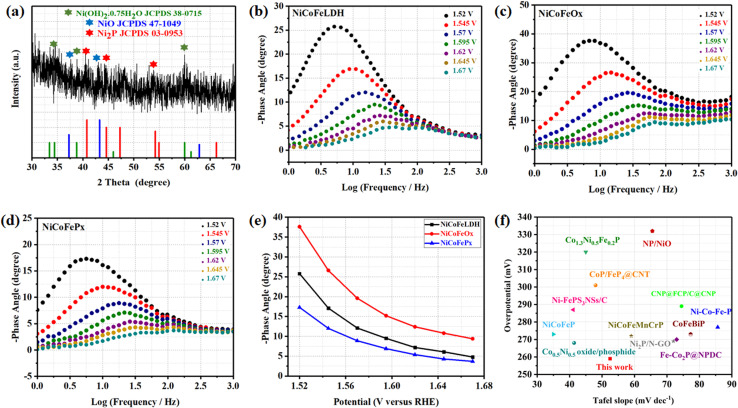
(a) XRD of NiCoFeP_*x*_ after OER; Bode plots at range from 1.52 to 1.67 V (*vs.* RHE) of (b) NiCoFeLDH; (c) NiCoFeO_*x*_; (d) NiCoFeP_*x*_; (e) potential dependence of the phase angle; (f) comparison of *η*_10_ overpotential and Tafel slope of NiCoFeP_*x*_ PNSs with some advanced NiCoFe-based OER electrocatalysts.

The surface morphology of post OER NiCoFeP_*x*_ PNSs catalyst was investigated using SEM and TEM images (Fig. S19, ESI†), which revealed the surface morphology of nanosheets didn't change during 1000 cycles of CV. Furthermore, the structure of post OER NiCoFeP_*x*_ PNSs catalyst was analyzed using HRTEM imaging (Fig. 5a), which revealed a heterostructure consisting of crystalline and amorphous regions.^34^ The crystalline region has lattice fringes with an interplanar spacing of 0.221 nm, which corresponds to the (111) crystal plane of Ni_2_P (JCPDS No. 03-0953) (Fig. 5b–d), and still has abundant lattice defects. The amorphous region corresponds to the formation of MO_*x*_(OH)_*y*_ on the surface. HRTEM confirmed that the internal phosphide structure remains very well and the formation of an amorphous MO_*x*_(OH)_*y*_ layer on the surface. The SAED image (Fig. 5e) and the inset FFT image refer to polycrystalline materials that possess a low-crystalline structure, and there are no complete diffraction rings but scattered light spots. The post OER HAADF STEM (Fig. 5f) and elemental EDX mapping (Fig. 5g–m) showed the homogeneous distribution of Ni, Co, Fe, P, O and C elements through the whole surface of NiCoFeP_*x*_ PNSs. Furthermore, the chemical composition of post OER NiCoFeP_*x*_ PNSs catalyst was investigated by XRD (Fig. 6a). The results showed that the intensity of phosphide peaks decreased with the appearance of some low intensity peaks of NiO and Ni(OH)_2_, which agree with the formation of an amorphous MO_*x*_(OH)_*y*_ layer on the surface in HRTEM. The results clarify that the catalysts undergo electrochemical surface reconstruction to form oxyhydroxide hybrid active sites, which consider the actual active sites, and Ni_2_P considers the pre-catalysts for OER.^35^ The heterostructure formed during the electrochemical activation could have better catalytic activity due to the abundance of active sites and modulation of the electronic structure. Fig. 6b–d exhibits the Bode plots of NiCoFeLDH, NiCoFeO_*x*_ and NiCoFeP_*x*_. Significant response signals in the high-frequency region corresponding to the oxidation of Ni^2+^ to Ni^3+^ are observed. The potential dependence of the phase angle (Fig. 6e) exhibited that NiCoFeP_*x*_ has the smallest phase angle at all voltages, which refers to the large number of electrons involved in the OER and the ease of adsorption and desorption between the active sites and the intermediates, which may be caused by electrochemical surface reconstruction.^36^ Also, the NiCoFeP_*x*_ PNSs exhibit not only excellent long-term stability but also superior electrocatalytic activity when compared to other NiCoFe-based OER catalysts reported in recent years (Fig. 6f and Table S3, ESI†).

**Fig. 7:**
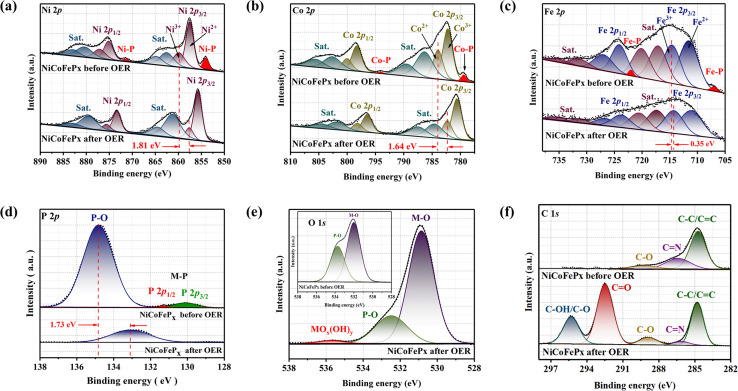
XPS fine spectra of NiCoFeP_*x*_ PNSs before and after OER: (a) high resolution Ni 2p; (b) high resolution Co2p; (c) high resolution Fe 2p; and (d) high resolution P 2p; (e) high resolution O 2p; (f) high resolution C 1s.

The XPS spectrum of the initial and post-OER of NiCoFeP_*x*_ PNSs catalysts after 1000 cycles was also studied to investigate the change in the electronic structure and the electrochemical surface reconstruction (Fig. S20, ESI†). The Ni 2p XPS profile (Fig. 7a) reveals a negative shift of Ni^3+^ binding energy, estimated to be 1.81 eV and the two peaks of Ni–P disappeared after OER. The Co 2p XPS profile (Fig. 7b) shows decreasing the intensity of the Co^2+^ peak and increasing the intensity of the Co^3+^ peak with a negative shift in binding energy estimated by 1.64 eV, which increases the high oxidation state Co^3+^ and decreases the lower oxidation state Co^2+^, and the two peaks of Co–P disappeared after OER. The Fe 2p XPS profile (Fig. 7c) shows a negative change in binding energy equal to 0.35 eV and the disappearance of the Fe–P peaks. The P 2p XPS profile (Fig. 7d) showed a negative shift by 1.73 eV of P–O with a decrease in peak intensity, and the M–P peaks disappeared.^37^ The O 1s (Fig. 7e) also showed a decrease in the intensity of P–O peak with a negative shift of binding energy, and a new peak of MO_*x*_(OH)_*y*_ appears after OER, which proves the electrochemical surface reconstruction.^38^ C 1s XPS profile (Fig. 7f) is another piece of evidence of the surface reconstruction. The appearance of two new peaks at around 292.5 and 295 eV after the OER can be attributed to the chemical changes that occur on the catalyst surface during the reaction. The peak at around 292.5 eV typically corresponds to C=O bonds, indicating the presence of carbon–oxygen species on the catalyst surface. This peak can be attributed to the adsorption of oxygen-containing species such as hydroxyls onto the catalyst surface during the OER. The peak at around 295 eV is often associated with C–OH or C–O bonds, indicating the presence of carbon–hydroxyl or carbon–oxygen species on the catalyst surface.^39^ By utilizing XPS data, we can investigate the electronic structure of the NiCoFeP_*x*_ PNSs catalyst before and after the formation of MO_*x*_(OH)_*y*_ active species. Shedding light on the charge transfer pathways between the phosphide and surface species. The disappearance of M−P peaks during activation refers to the fact that metal phosphides are usually electrochemically oxidized into P-containing species (phosphates PO_4_^3−^, PO_3_^−^, or P_2_O_5_), which is also the main mode of P–O binding, which dissolves in alkali and facilitates the electrochemical surface reconstruction, and generates MO_*x*_(OH)_*y*_ active sites and facilitates the electron transfer during the OER.^40^ The charge transfers between phosphides and MO_*x*_(OH)_*y*_ are very important for enhancing catalytic activity, electron transfer, and long-term stability. The exceptional electrocatalytic activity of the NiCoFeP_*x*_ PNSs can be attributed to their unique properties, such as mesoporous nanosheets with high ECSA, a high density of defects, and active sites resulting from electrochemical surface reconstruction, which lead to excellent stability and durability in alkaline conditions.

## 4. Conclusion

In summary, the NiCoFe-based phosphides have been prepared by a simple one-step hydrothermal reaction using two different mixtures of solvents (oleylamine, ethanol and water) and (urea, ammonium fluoride and water) and chemical vapor deposition-based phosphorization. The NiCoFeP_*x*_ PNSs morphology revealed mesoporous ultrathin nanosheets with a low crystalline surface and abundant active sites, but the NiCoFeP_*x*_-W PNSs revealed hexagonal nanosheets with a crystalline structure. The low crystalline NiCoFeP_*x*_ PNSs reveal the best electrocatalytic activity, which exhibits an overpotential of only 259 mV at 10 mA cm^−2^, a small Tafel slope of 50.47 mV dec^−1^ and excellent electrochemical durability in alkaline. The superior electrocatalytic activity of the NiCoFeP_*x*_ PNSs can be attributed to their morphology with mesoporous nanosheets with a high density of lattice defects and active sites, a high electrochemically active surface area (ECSA) caused by electrochemical surface reconstruction and the formation of MO_*x*_(OH)_*y*_ active sites, and efficient charge transfer dynamics. These advantages ensure that the NiCoFeP_*x*_ PNSs electrocatalysts have good application prospects in practical electrochemical water splitting. This work provides a new path for optimizing metal phosphide electrocatalysts.

## Conflicts of interest

There are no conflicts to declare.

## Supplementary Material

RA-014-D4RA02344G-s001
