# Supplementary material for: Electrochemical water splitting enhancement by introducing mesoporous NiCoFe-trimetallic phosphide nanosheets as catalysts for the oxygen evolution reaction
Source: RSC Adv. 2024 May 28;14(24):17202–12. doi: 10.1039/d4ra02344g (PMC11132062; doi:10.1039/d4ra02344g)
Supplement: RA-014-D4RA02344G-s001 [file RA-014-D4RA02344G-s001.pdf]

Electronic Supplementary Material (ESI) for RSC Advances.
This journal is © The Royal Society of Chemistry 2024

Electronic Supplementary Information

Electrochemical water splitting enhancement by introducing mesoporous NiCoFe-trimetallic phosphide nanosheets as catalysts for the oxygen evolution reaction

Gouda Helal,[ǂab] Zhenhang Xu,[ǂa] Wei Zuo,[a] Yueying Yu,[c] Jinyan Liu,*[d] Hongping Su,[e] Jianxin Xu,[e] Houbin Li,[c] Gongzhen Cheng *[a] and Pingping Zhao*[c]

Supplementary Images characterization:

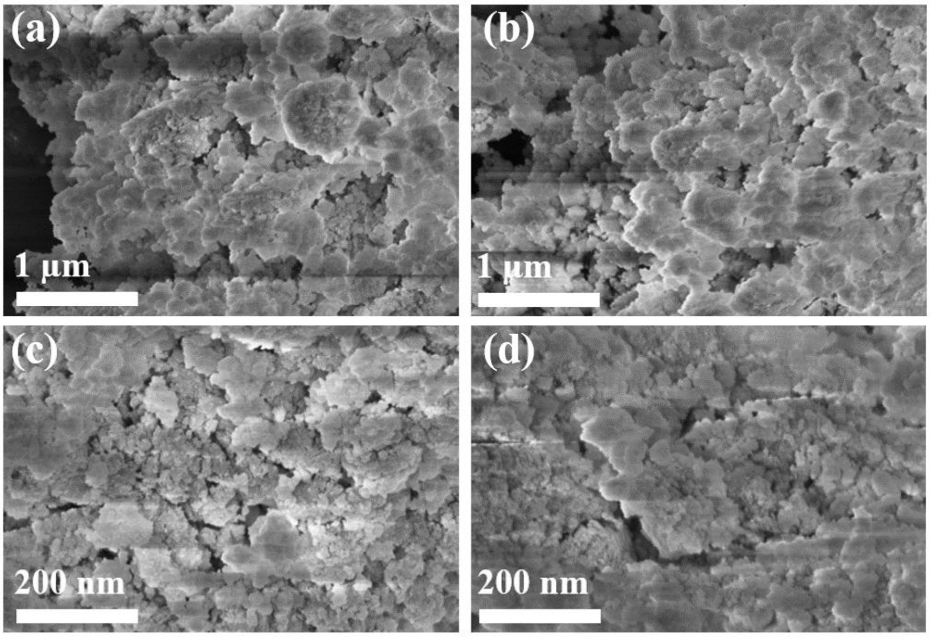

Fig. S1: (a-d) SEM images of NiCoFeLDH PNSs prepared using solvents (OAM, Ethanol, and Water).

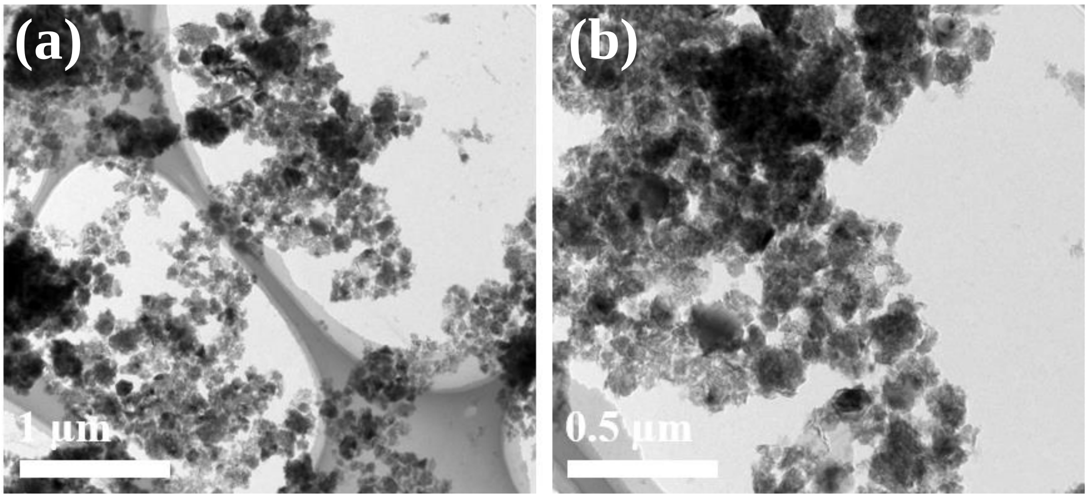

Fig. S2: (a-b) TEM images of NiCoFeLDH PNSs prepared using solvents (OAM, Ethanol, and Water).

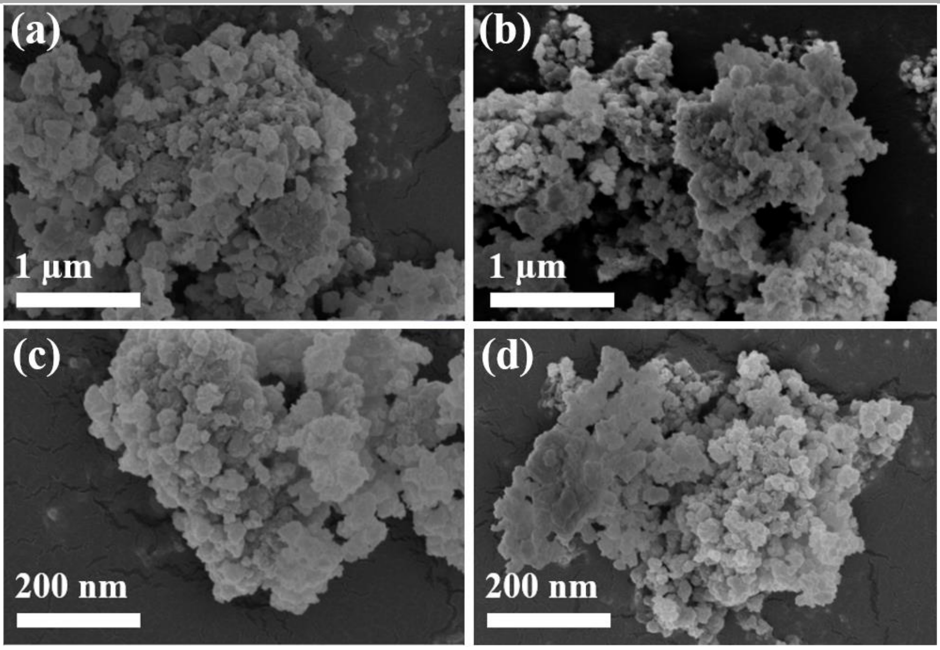

Fig. S3: (a-d) SEM images of NiCoFeOx PNSs prepared using solvents (OAM, Ethanol, and Water).

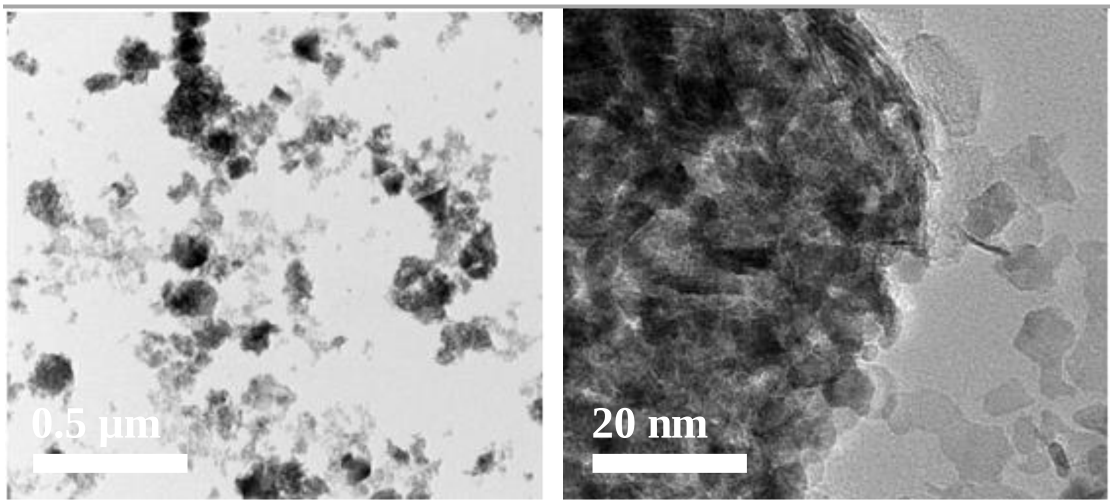

Fig. S4: (a-b) TEM images of NiCoFeOx PNSs prepared using solvents (OAM, Ethanol, and Water).

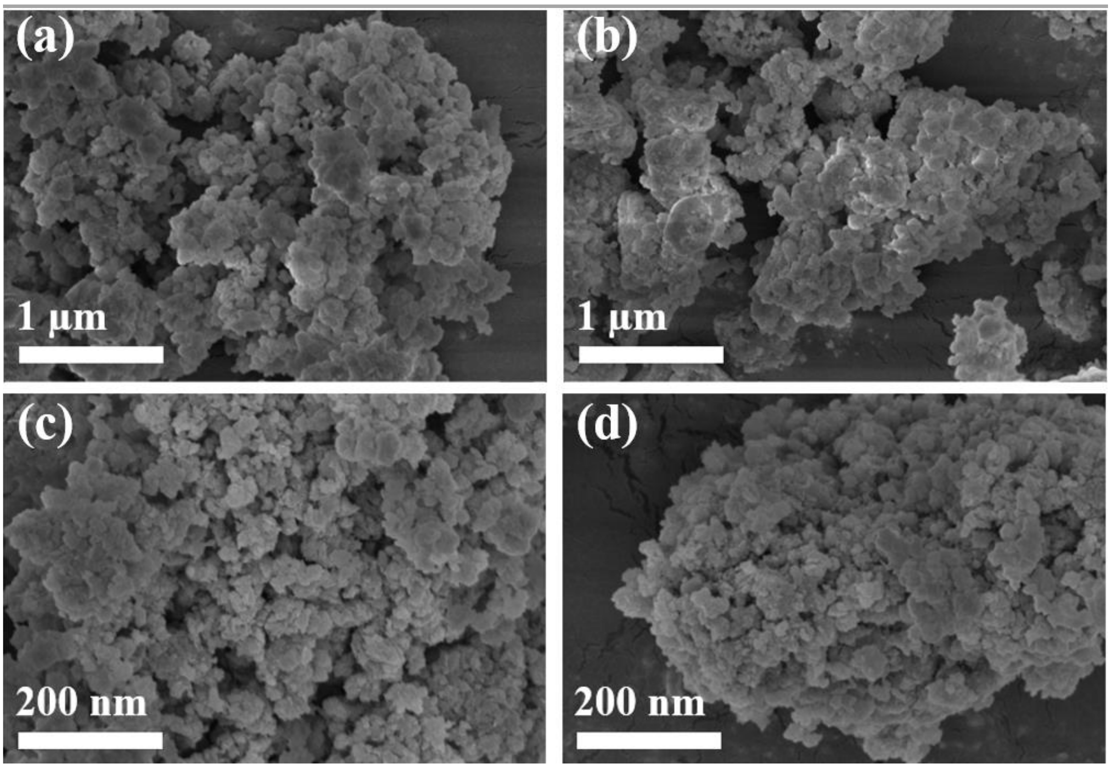

Fig. S5: (a-d) SEM images of NiCoFePx PNSs prepared using solvents (OAM, Ethanol, and Water).

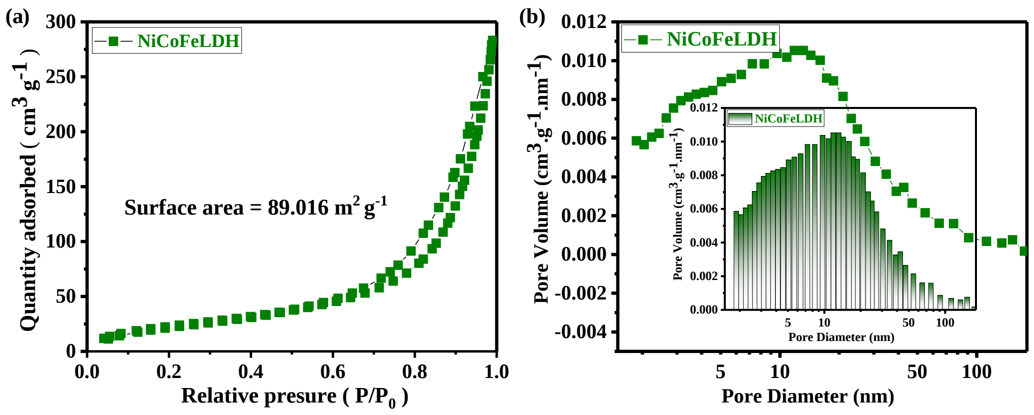

Fig. S6: (a) N_2 adsorption desorption of NiCoFeLDH PNSs prepared using solvents (OAM, Ethanol, and Water); (b) Pore size distribution curve corresponding to specific pore volume of NiCoFeLDH PNSs.

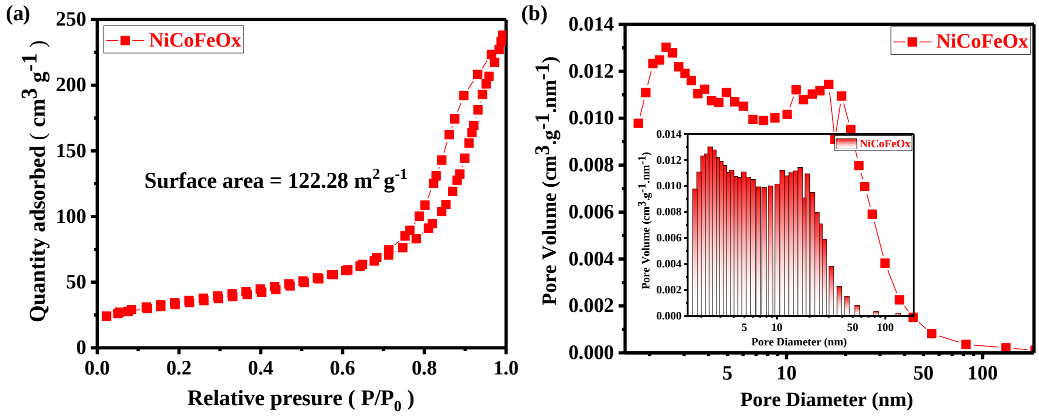

Fig. S7: (a) N_2 adsorption desorption of NiCoFeOx PNSs prepared using solvents (OAM, Ethanol, and Water); (b) Pore size distribution curve corresponding to specific pore volume of NiCoFeOx PNSs.

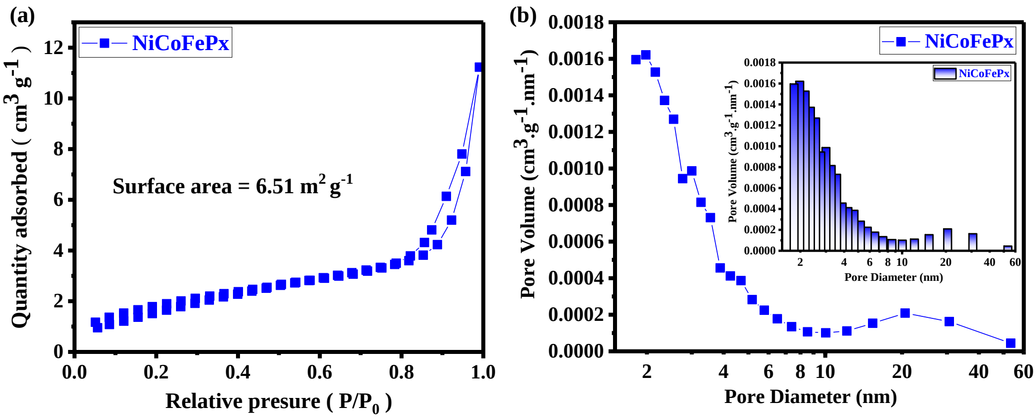

Fig. S8: (a) N_2 adsorption desorption of NiCoFePx PNSs prepared using solvents (OAM, Ethanol, and Water); (b) Pore size distribution curve corresponding to specific pore volume of NiCoFePx PNSs.

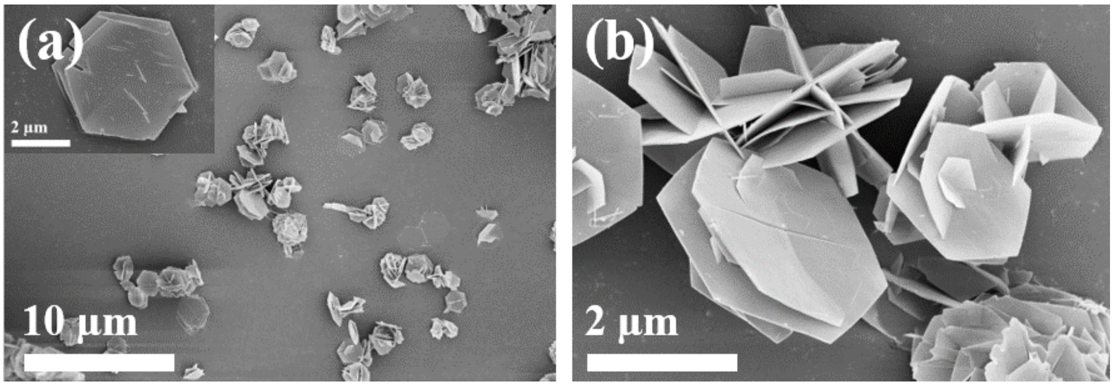

Fig. S9: (a-b) SEM images of NiCoFeLDH-W prepared using solvents (Urea, Ammonium fluoride and Water).

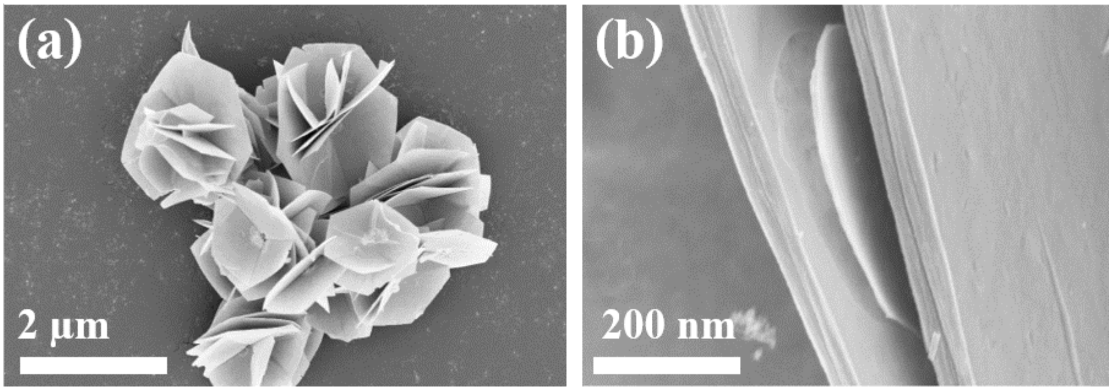

Fig. S10: (a-b) SEM images of NiCoFeOx-W prepared using solvents (Urea, Ammonium fluoride and Water).

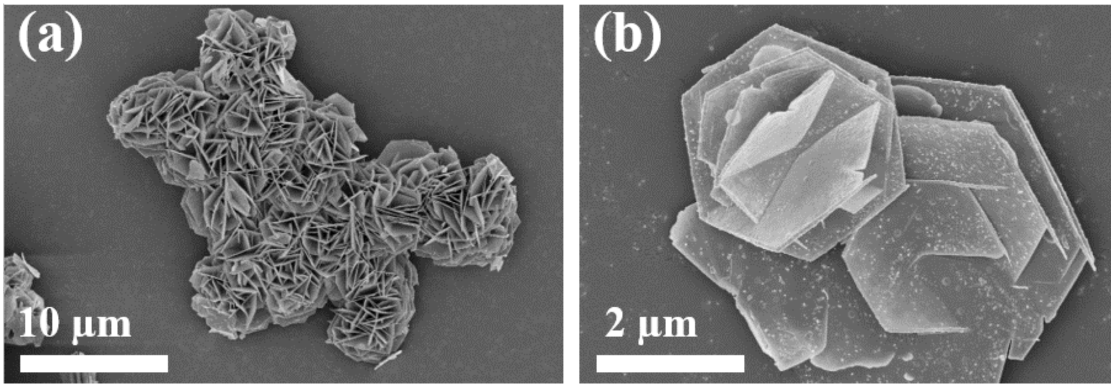

Fig. S11: (a-b) SEM images of NiCoFePx-W prepared using solvents (Urea, Ammonium fluoride and Water).

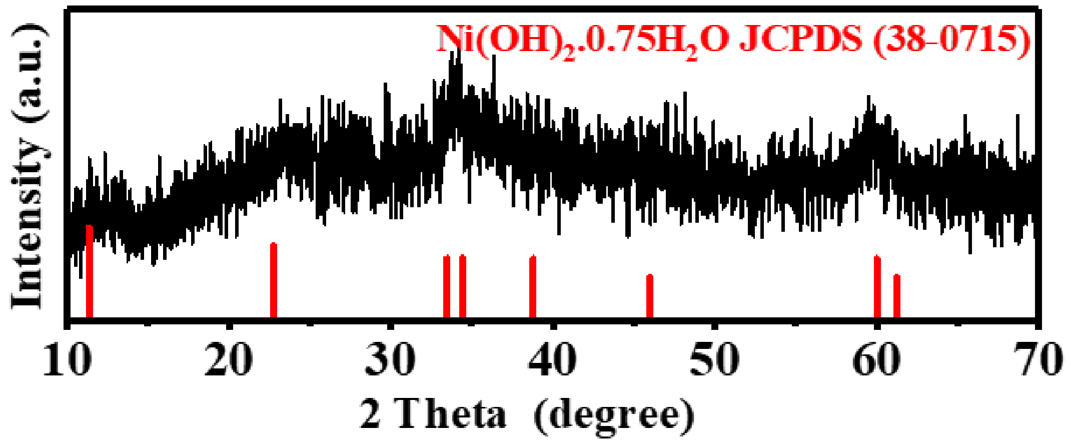

Fig. S12: XRD image of NiCoFeLDH PNSs prepared using solvents (OAM, Ethanol, and Water).

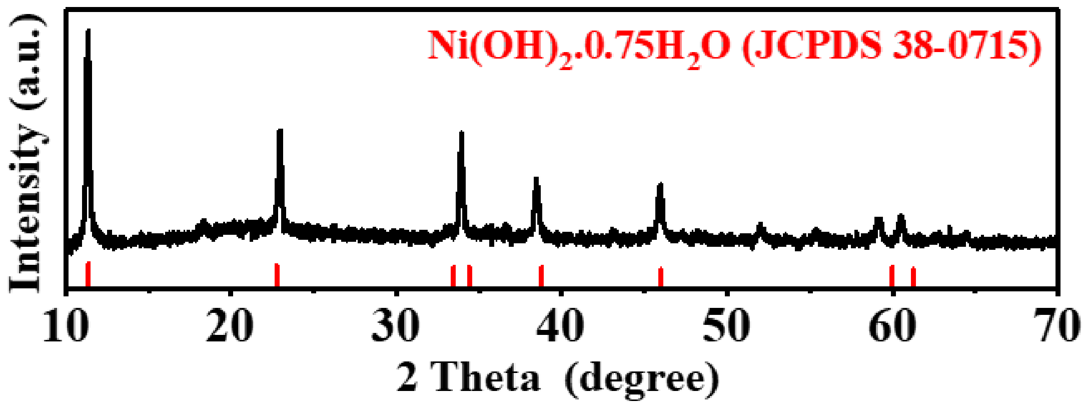

Fig. S13: XRD image of NiCoFeLDH-W prepared using solvents (Urea, Ammonium fluoride and Water).

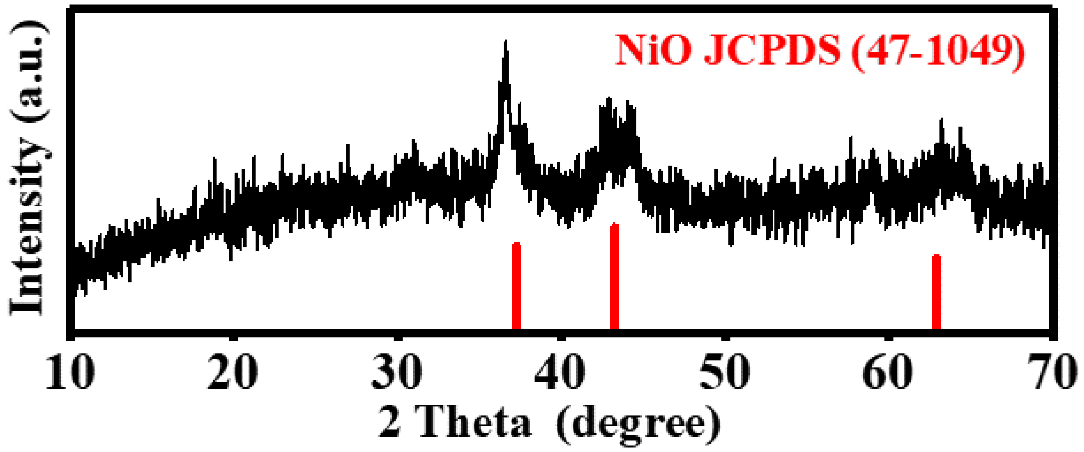

Fig. S14: XRD image of NiCoFeOx PNSs prepared using solvents (OAM, Ethanol, and Water).

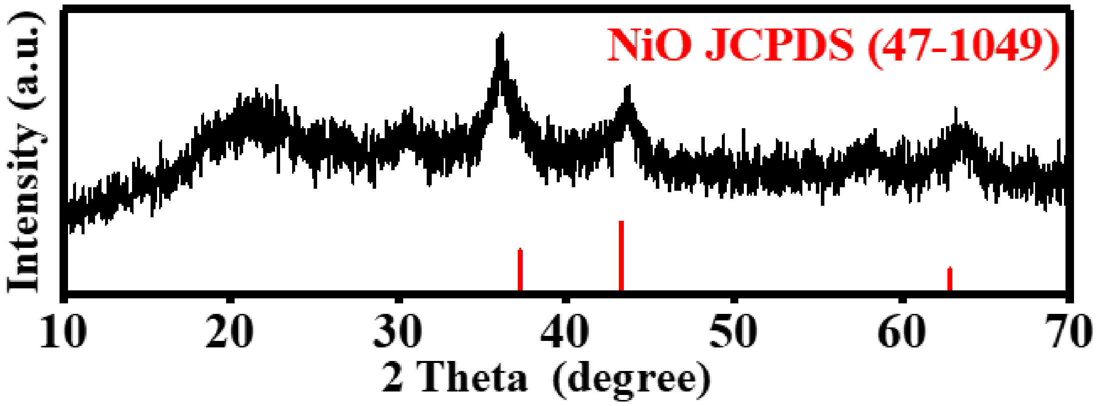

Fig. S15: XRD image of NiCoFeOx-W prepared using solvents (Urea, Ammonium fluoride and Water).

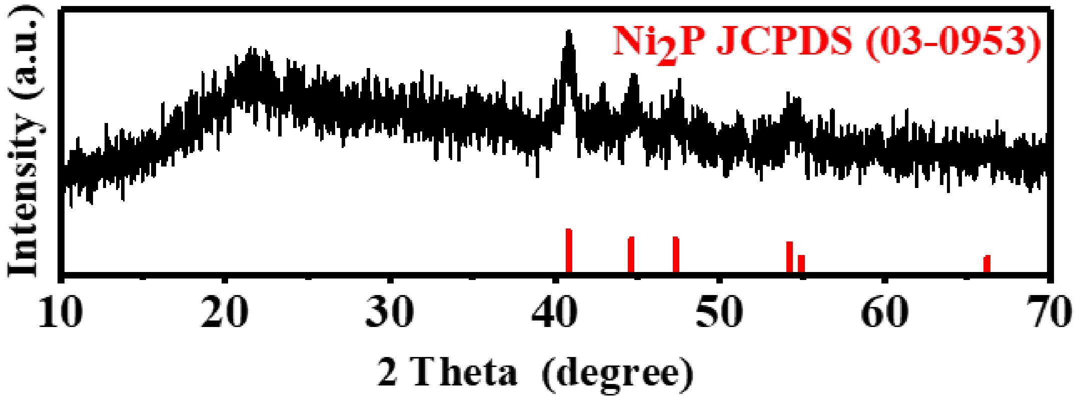

Fig. S16: XRD image of NiCoFePx-W prepared using solvents (Urea, Ammonium fluoride and Water).

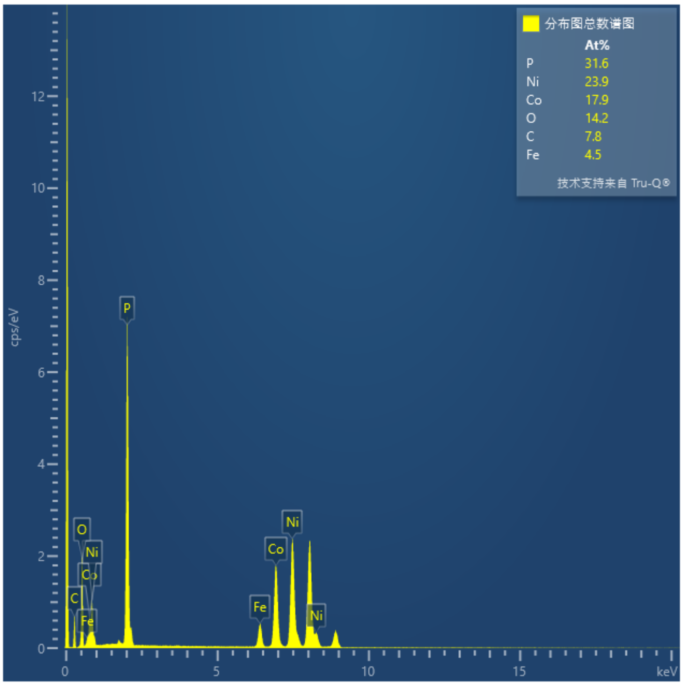

Fig. S17: EDX spectra of NiCoFePx prepared using solvents (OAM, ethanol and Water).

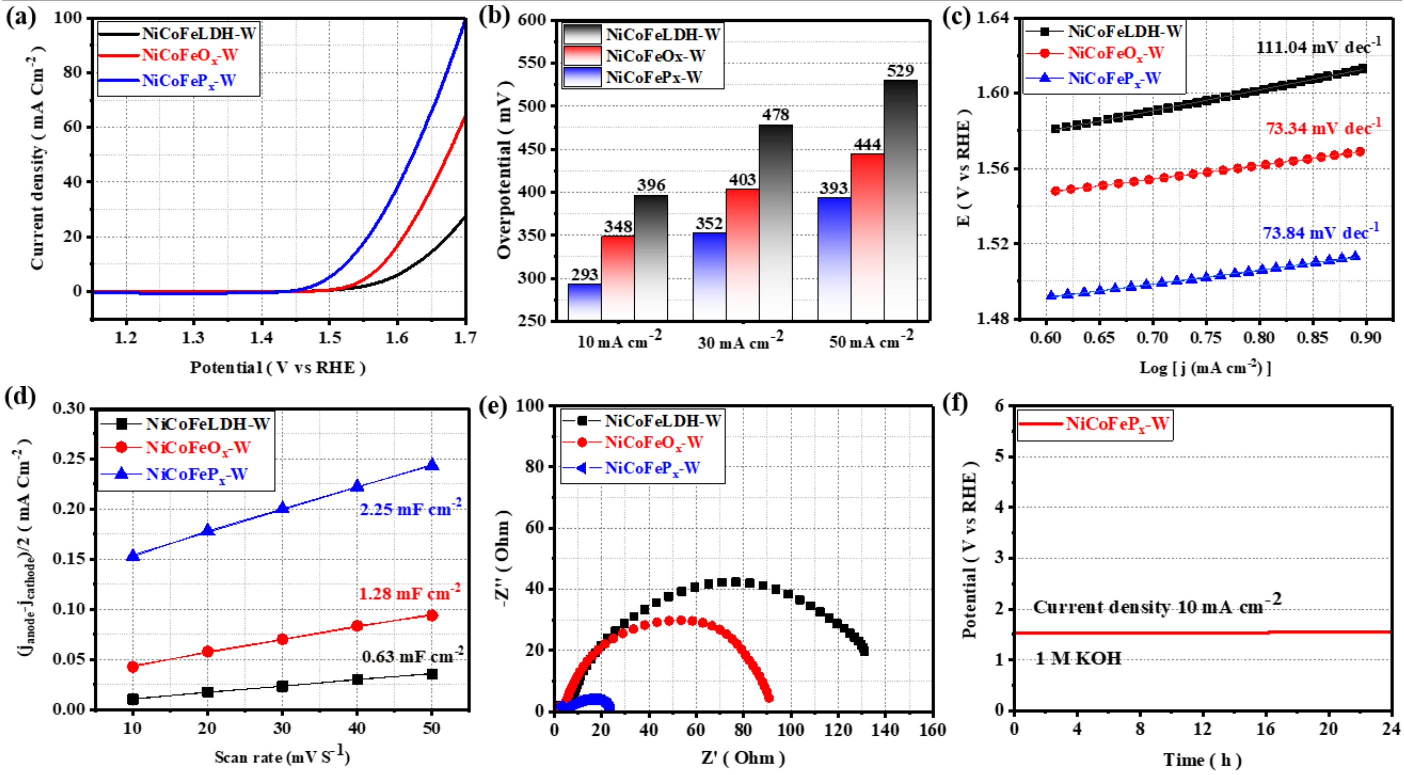

Fig. S18: Electrochemical measurement results of NiCoFeLDH-W, NiCoFeOx-W and NiCoFePx-W prepared using solvents (Urea, Ammonium fluoride and Water): (a) LSV curves of OER; (b) Overpotential histogram at 10, 30 and 50 mA cm^{-2}; (c) Tafel plots; (d) C_{dl} values; (e) Nyquist plots measured at 0.6 V versus Hg/HgO; (f) Durability test of NiCoFePx-W at current density 10 mA cm^{-2}.

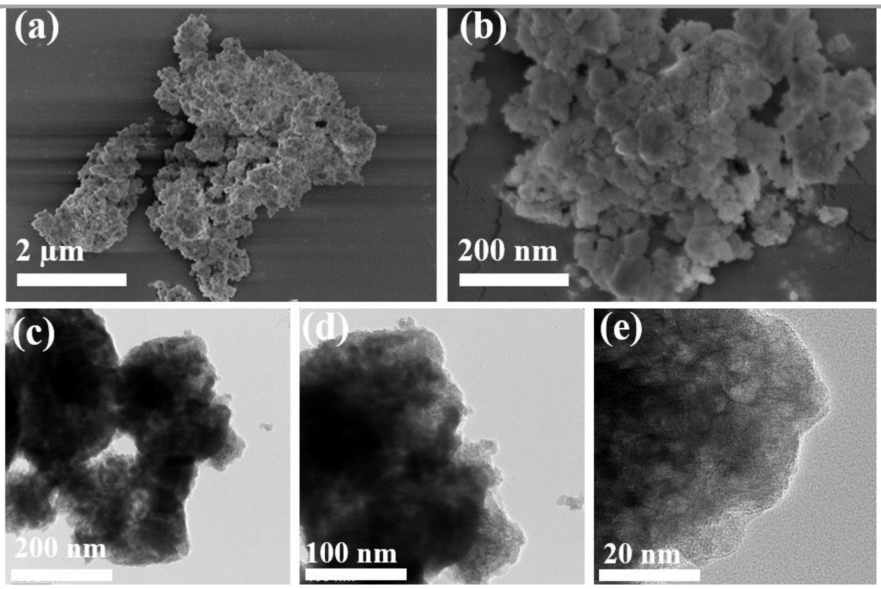

Fig. S19: Surface morphology of NiCoFePx PNSs prepared using solvents (OAM, Ethanol, and Water) after OER: (a-b) SEM image; and (c-e) TEM image.

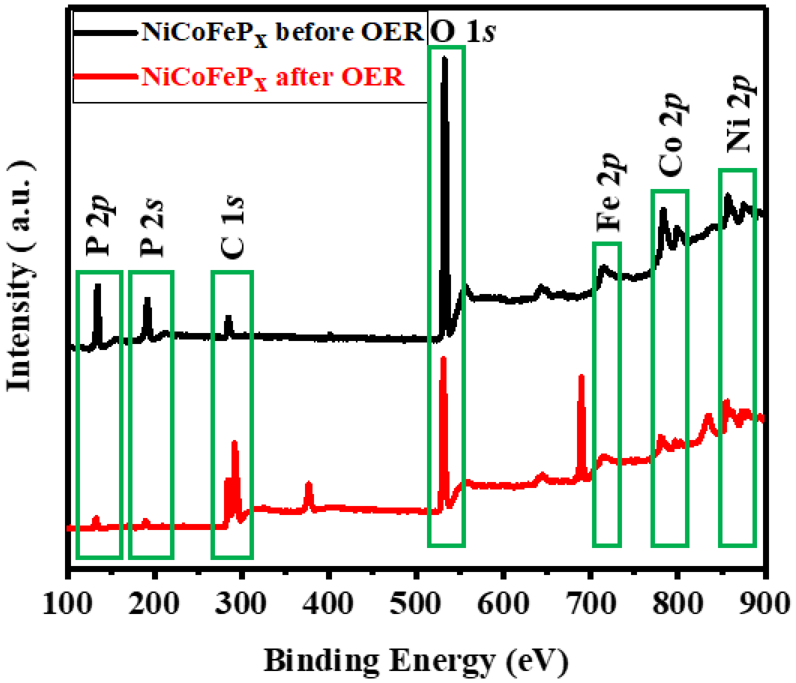

Fig. S20: XPS spectra of NiCoFePx PNSs prepared using solvents (OAM, Ethanol, and Water) after OER.

Supplementary Tables:

Table S1: The BET and BJH data.

Sample	BET ($m^2\ g^{-1}$)	BJH ($cm^3\ g^{-1}$)	Pore size (nm)
**NiCoFeLDH**	**89.016**	**0.43**	**14.75**
**NiCoFeOx**	**122.28**	**0.37**	**10.99**
**NiCoFePx**	**6.51**	**0.017**	**10.67**

Table S2: The metal elements composition (%) of samples determined by ICP.

sample	Ni	Co	Fe
**NiCoFeLDH**	**22.94**	**17.44**	**4.58**
**NiCoFeOx**	**39**	**29.42**	**7.8**
**NiCoFePx**	**27.42**	**22.38**	**5.66**

Table S3: Comparison of OER activity of NiCoFePx and NiCoFePx-W nanosheets in this work with other reported electrocatalysts in 1 M alkaline solution.

Catalyst	η_{10mA/cm^2} (mV)	Tafel slope (mV dec^{-1})	Stability	Reference
**NiCoFePx**	259	52.48	24 h at 10 mA cm^{-1}	This work
**NiCoFePx-W**	293	73.84	24 h at 10 mA cm^{-1}	This work
**CNP@FCP/C@CNP**	289	74.5	24 h at 10 mA cm^{-1}	[1]
**Ni-Co-Fe-P**	277	85.64	24 h at 20mA cm^{-1}	[2]
**Co1.3Ni0.5Fe0.2P**	320	45	10 h at 10mA cm^{-1}	[3]
**Fe-Co_2P@NPDC**	270	73	24 h at 10mA cm^{-1}	[4]
**Ni-$FePS_3$ NSs/C**	287	41.1	48 h at 10mA cm^{-1}	[5]
**NiCoFeP**	273	35	10 h at 273 mV	[6]
**Ni_2P/N-GO**	269	72	7h at 0.45 V	[7]
**NP/NiO**	332	65.5	15 h at 10 mA cm^{-1}	[8]
**CoP/FeP_4@CNT**	301	48	20 h at 1.533 V	[9]
**CoFeBiP**	273	77.3	25 h at 10 mA cm^{-1}	[10]
**NiCoFeMnCrP**	272	59	24 h at 10 mA cm^{-1}	[11]
**$Co_{0.5}Ni_{0.5}$ oxide/phosphide**	268	41.4	24 h at 10 mA cm^{-1}	[12]

References

[1] J. Shi, W. Peng, Y. F. Yang, B. Li, J. Nie, H. Wan, Y. Li, G. F. Huang, W. Hu, W. Q. Huang, Small., 2023, 19, 2302906.

[2] Y. Wang, Y. Wang, H. Gao, Z. Huang, Q. Hao, B. Liu, Chem. Eng. J., 2023, 451, 138515.

[3] J. Chen, J. Ying, Y. Xiao, Y. Dong, K. I. Ozoemena, S. Lenaerts, X. Yang, Sci. China Mater., 2022, 65, 2685-2693.

[4] L. Li, L. Zhang, Z. Nie, W. Ma, N. Li, T. Wågberg, G. Hu. J. Mater, Chem. A., 2022, 10, 21659-21671.

[5] C. Tang, D. He, N. Zhang, X. Song, S. Jia, Z. Ke, J. Liu, J. Wang, C. Jiang, Z. Wang, Energy Environ. Mater., 2022, 5, 899-905.

[6] Y. Guo, J. Tang, Z. Wang, Y. Sugahara, Y. Yamauchi, Small., 2018, 14, 1802442.

[7] M. Yang, R. Zhao, Y. Liu, H. Lin, CrystEngComm., 2022, 24, 1189-1194.

[8] P. Bhanja, Y. Kim, B. Paul, Y. V. Kaneti, A. A. Alothman, A. Bhaumik, Y. Yamauchi, Chem. Eng. J., 2021, 405, 126803.

[9] Y. Liu, Y. Li, Q. Wu, Z. Su, B. Wang, Y. Chen, S. Wang, Nanomaterials., 2021, 11, 1450.

[10] C. Wang, H. Shang, Y. Wang, J. Li, S. Guo, J. Guo, Y. Du, Nanoscale., 2021, 13, 7279-7284.

[11] D. Lai, Q. Kang, F. Gao, Q. Lu. J. Mater, Chem. A., 2021, 9, 17913-17922.

[12] Y. Yang, H. Wan, G. Chen, N. Zhang, J. Li, W. Ma, X. Liu, R. Ma, Dalton Trans., 2020, 49, 10918-10927.
